# Use of drugs for gastrointestinal disorders: evidence from National Survey on Access, Use and Promotion of Rational Use of Medicines

**DOI:** 10.31744/einstein_journal/2020AO5314

**Published:** 2020-08-06

**Authors:** Lucas Borges Pereira, Ana Maria Rosa Freato Gonçalves, Camila Stéfani Estancial Fernandes, Andréia Turmina Fontanella, Priscila Maria Stolses Bergamo Francisco, Sotero Serrate Mengue, Rogério Boff Borges, Tatiane da Silva Dal Pizzol, Karen Sarmento Costa

**Affiliations:** 1 Faculdade de Ciências Farmacêuticas de Ribeirão Preto Universidade de São Paulo Ribeirão PretoSP Brazil Faculdade de Ciências Farmacêuticas de Ribeirão Preto, Universidade de São Paulo, Ribeirão Preto, SP, Brazil.; 2 Departamento de Saúde Coletiva Faculdade de Ciências Médicas Universidade Estadual de Campinas CampinasSP Brazil Departamento de Saúde Coletiva, Faculdade de Ciências Médicas, Universidade Estadual de Campinas, Campinas, SP, Brazil.; 3 Programa de Pós-Graduação em Epidemiologia Faculdade de Medicina Universidade Federal do Rio Grande do Sul Porto AlegreRS Brazil Programa de Pós-Graduação em Epidemiologia, Faculdade de Medicina, Universidade Federal do Rio Grande do Sul, Porto Alegre, RS, Brazil.

**Keywords:** Drug utilization, Pharmaceutical services, Gastrointestinal tract, Health surveys, Pharmacoepidemiology, Proton pump inhibitors, Polypharmacy, Aged, Health policy, Patient medication knowledge

## Abstract

**Objective:**

To estimate the prevalence of use of drugs to treat gastrointestinal disorders, according to demographic, socioeconomic, and health characteristics of the Brazilian population.

**Methods:**

This is a population-based survey that interviewed individuals residing in cities of the five regions in Brazil. The study sample was composed of 32,348 individuals aged 20 or more years. The profile of use of drugs for gastrointestinal disorders was evaluated considering the variables sex, age, healthcare plan, region, and number of chronic diseases. We also analyzed the frequency of individuals who declared using other drugs, besides those already employed for treatment of gastrointestinal disorders. Additionally, the estimated frequencies of the drug classes used were determined.

**Results:**

The prevalence of use of drugs for gastrointestinal disorders in Brazil was 6.9% (95% confidence interval − 6.4-7.6), higher in females, among persons aged over 60 years, in those who had a private healthcare insurance, and presented with two or more chronic diseases. It was noted that 42.9% of the aged who used drugs for gastrointestinal disorders were also on polypharmacy. As to the classes of drugs, 82% corresponded to drugs for the food tract and metabolism, particularly proton pumps inhibitors.

**Conclusion:**

The use of drugs for treatment of gastrointestinal disorders was significant among women and elderly. In this age group, consumption may be linked to gastric protection due to polypharmacy. This study is an unprecedented opportunity to observe the self-reported consumption profile of these drugs in Brazil and, therefore, could subsidize strategies to promote their rational use.

## INTRODUCTION

Gastrointestinal disorders (GID) are characterized by signs and symptoms that affect tissues and organs of the gastrointestinal tract, such as nausea, abdominal pain, and a burning sensation originated from an underlying disease.^( 1 )^Approximately 27% of world population has constipation, one of the various disorders that can affect the gastrointestinal tract.^( 2 )^Additionally, other factors can lead to the appearance of these disorders, such as the use of some drugs,^( 3 )^ eating, and lifestyle.^( 4 )^In this way, the use of drugs for the relief of these disorders becomes frequent in the population.

Instead of investigating the underlying disease or external factors that can originate the symptoms, the majority of patients prefer to use drugs that bring relief. One of the main reasons is the ease of buying these drugs, since most are sold over the counter (OTC).^( 5 )^ Also, in a study carried out in the United States, 34% of physicians were not concerned with or ignored the potential problems these drugs could cause to the patient.^( 6 )^

Despite the OTC having safer characteristics when compared to other drugs, many of them can cause mild to severe adverse events, especially when taking into consideration the physiological particularities and other characteristics, such as age group, pregnancy, and the use of even more drugs.^( 3 )^ Additionally, approximately 70% of pregnant women in the world experience nausea, and 50% present vomiting;^( 3 )^ 8% of medication errors in nursing homes are caused by drugs to treat GID;^( 7 )^70.8% of patients who are on polypharmacy use gastric protection drugs.^( 8 )^ In this setting, special attention is needed for these populations, with the objective that drugs for GID be used in a rational manner, with the purpose of reducing risks.

Generally speaking, the scientific literature presents drug utilization researches to treat GID that focus on the analyses of the subclasses of these drugs, such as H2 receptor antagonists and proton pump inhibitors (PPI),^( 9 )^ specific health conditions,^( 10 , 11 )^and in specific populations.^( 4 )^

In Brazil, the largest survey study conducted about GID was the EpiGastro (2014), carried out in the city of São Paulo. In this study, 3,050 people were interviewed about information that could be associated with gastroesophageal reflux and dyspepsia, as well as how this population dealt with the symptoms. It was noted that 13.6% did not use drugs to treat symptoms, and 34.2% used drugs not prescribed by physicians.^( 11 )^ However, there are no detailed analyses about drugs to treat GID. Thus, there are few studies that broadly evaluate the use of these drugs in large populations.

Considering the importance of the topic and the scarcity of population-based studies, is it vital to develop surveys that trace the profile of use of these drugs in the different population segments, in order to prepare strategies for their rational use.

## OBJECTIVE

To estimate the prevalence of use of drugs for the treatment of gastrointestinal disorders, as per health, demographic and socioeconomic characteristics of the Brazilian population; to analyze the frequency of use of other medicines concomitant to the drugs to treat gastrointestinal disorders; and to describe the classes of most used drugs for the treatment of gastrointestinal disorders.

## METHODS

This study has a cross-sectional population-based design; it was conducted using data from the National Survey on Access, Use, and Promotion of Rational Use of Medicines (PNAUM - *Pesquisa Nacional sobre o Acesso, Utilização e Promoção do Uso Racional de Medicamentos* ). Data collection was performed between September 2013 and February 2014, in which 41,433 individuals were interviewed, distributed over 245 municipalities of all regions of the country. The complete methodology of this survey is available at Mengue et al.^( 12 )^

This analysis covers individuals aged 20 or more years, residing in urban areas, and capable of communicating (n=32,348). The use of drugs for GID was evaluated by means of the question: “over the last 15 days, did you take any medicine for stomach or intestinal problems?”

Analyses were made using Stata 11.0 (StataCorp LP, College Station, Texas, USA), whose procedures for analysis of populational surveys incorporate aspects of the complex sample, by means of the SVY commands. Stratified analyses were made for two age groups: adults (20 to 59 years) and elderly (60 or more years). The 60-year classification for elederly classification was chosen in accordance with the Senior Citizen Statute.^( 13 )^For each age group, an estimate was made of the prevalence of use of drugs for GID, according to demographic (sex and region of Brazil where the patient resides), socioeconomic (holder of a private health insurance), and health (number of chronic diseases) characteristics. The association between the use of drugs for GID and the independent variables was verified by means of the χ^2^ test for homogeneity, with a 5% significance level.

In order to understand the influence of polypharmacy on the use of drugs to treat GID, the frequency, percentage, and respective 95% confidence interval (95%CI) were calculated for individuals who declared the use, in addition to drugs for GID, of no other drug, one or two drugs, three or four drugs, or five or more drugs. In this study, this last category was considered as an individual on polypharmacy.^( 14 )^

Frequencies, percentages, and respective 95%CI of drug classes used were identified and estimated according to the first category of the ATC. The substances that did not fit in the ATC classification were categorized as non-classifiable by ATC combinations, and were composed of combinations of drugs with the purpose of acting on different mechanisms that converge towards the same result, but are not classified by ATC. For example, choline citrate + betaine + methionine indicated to treat metabolic or hepatic disorders, and caffeine + dipyrone + orphenadrine, indicated for the relief of pain associated with muscle contractures or tension headaches); plants/phytotherapicsherbal medicines, composed of plants, teas, bottled substances, dyes, and herbal medicines; homeopathics, and when they could not be identified, were labeled as “non-identified” (these were the drugs entered in the questionnaire, but which cannot be identified, likely due to errors in incorrect typing of the drug).

National Survey on Access, Use, and Promotion of Rational Use of Medicines was approved by the National Ethics in Research Comission, opinion 398.131, CAAE: 18947013.6.0000.0008. All participants signed the Informed Consent Form (ICF).

## RESULTS

The prevalence of use of drugs for GID in the adult Brazilian population was 6.9% (95%CI: 6.4-7.6), higher among women. Considering the age groups for both sexes, the prevalence in the elderly was double that observed among adults (14.4% *versus* 7.1% in women, and 8.9% *versus* 4.2% in men, respectively). Also noted was the higher prevalence of use of these drugs by those who had a health insurance (8.5%; 95%CI: 7.2-10.0%) relative to those who did not (6.5%; 95%CI: 5.9-7.1) at the time of the study. The use of drugs to treat GID was 16.2% among the individuals who reported the presence of two or more chronic diseases and 3.8% (95%CI: 3.4-4.3) in those who presented with no chronic disease ( Table 1 ).

**Table 1:** Characteristics of the sample and prevalence of use of drugs for the treatment of gastrointestinal disorders in the Brazilian population according to demographic, socioeconomic, and health characteristics

Variable	Sample n (%)	Prevalence of use in the general population		Prevalence of use in adults (20-59 years)		Prevalence of use in the elderly (60 or more years)	
		
		%	95%CI	n (%)	95%CI	n (%)	95%CI
Total	32,348 (100.0)	6.9	6.4-7.6	23,283 (5.8)	5.2-6.4	8,995 (12.1)	10.9-13.4
Sex		p<0.001		p<0.001		p<0.001	
Female	20,646 (53.7)	8.6	7.8-9.4	15,351 (7.1)	6.4-7.9	5,246 (14.4)	12.8-16.3
Male	11,702 (46.3)	5.0	4.4-5.8	7,932 (4.2)	3.5-5.1	3,749 (8.9)	7.7-10.2
Health insurance		p<0.001		p<0.05		p=0.0887	
Yes	6,156 (23.6)	8.5	7.2-10.0	4,139 (7.0)	5.8-8.6	1,999 (13.9)	11.3-16.9
No	26,156 (76.4)	6.5	5.9-7.1	19,120 (5.4)	4.8-6.0	6,987 (11.4)	10.2-12.8
Region		p=0.1839		p=0.5159		p<0.003	
North	8,421 (6.7)	6.7	5.5-8.1	6,400 (6.4)	5.2-7.9	2,012 (8.5)	6.9-10.5
Northeast	6,909 (23.4)	6.3	5.4-7.2	4,839 (5.7)	4.8-6.7	2,054 (9.4)	8.0-11.0
Southeast	6,075 (47.5)	7.5	6.5-8.7	4,215 (6.0)	5.0-7.2	1,849 (13.2)	11.2-15.6
South	6,097 (14.7)	6.4	5.5-7.4	4,286 (5.0)	4.1-6.0	1,785 (12.4)	10.7-14.4
Mid-West	4,846 (7.8)	6.8	5.8-7.8	3,543 (5.5)	4.4-6.7	1,295 (13.4)	11.1-16.0
Number of chronic diseases		p<0.001		p<0.001		p<0.001	
None	17,972 (60.3)	3.8	3.4-4.3	15,779 (3.7)	3.2-4.2	2,162 (4.9)	3.9-6.3
One	7,380 (21.0)	7.8	6.8-8.9	4,620 (7.5)	6.5-8.7	2,743 (8.6)	6.6-11.1
Two or more	6,973 (18.7)	16.2	14.8-17.7	2,875 (15.1)	13.2-17.1	4,077 (17.4)	15.5-19.4

p value of the χ^2^ test. 95%CI: 95% confidence interval.

As to the use of other drugs in addition to those for the treatment of GID, monotherapy occurred in only 16.3% (95%CI: 13.9-19.0) of the general population, in which 20.7% (95%CI: 17.2-24.6) was in adults, and 7.3% (95%CI: 4.8-10.7) in the elderly. Among the aged, approximately 43.0% of those who used drugs for GID also reported using five or more drugs (polypharmacy) ( Table 2 ).

**Table 2:** Frequency of use of other drugs in the population that takes medicines for treatment of gastrointestinal disorders

Variable	General population		Adults (20-59 years)		Aged (60 or more years)		p value
		
	%	95%CI	%	95%CI	%	95%CI	
Number of other drugs							<0.001
None	16.3	13.9-19.0	20.7	17.2-24.6	7.3	4.8-10.7	
1-2	35.5	32.2-38.9	41.8	37.5-46.3	22.4	18.7-26.5	
3-4	22.2	20.1-24.5	19.7	16.9-22.8	27.4	24.1-31.0	
5 or more	26.0	23.5-28.5	17.8	15.0-21.0	42.9	37.8-48.1	

p value of the χ^2^ test. 95%CI: 95% confidence interval.

Table 3 shows the distribution of drug classes used for the treatment of GID. Approximately 82.0% (95%CI: 79.0-84.3) of drugs referred by the Brazilian population for treatment of GID were those classified as drugs for the alimentary tract and metabolism; 75.5% were drugs to treat peptic ulcers and gastroesophagic reflux disease (65.2% were PPI); 6.8% antacids; 5.9% for gastrointestinal function disorders; 4.2% propulsive; and 4.0% antiemetic and antinausea agents.

**Table 3:** Distribution of drug classes used to treat gastrointestinal disorders in the Brazilian population, as per age range

First ATC level	Pharmacologic classes as per ATC	General population		Adults (20-59 years)		Aged (60 or more years)	
		
		n (%)	95%CI	n (%)	95%CI	n (%)	95%CI
A	Alimentary tract and metabolism	1,981 (81.8)	79.0-84.3	1,156 (81.4)	77.7-84.6	825 (82.7)	78.7-86.1
J	Antiinfectives for systemic use	44 (2.2)	1.1-4.2	38 (3.0)	1.5-6.1	6 (0.4)	0.1-1.7
N	Nervous system	38 (1.3)	0.8-2.1	17 (0.9)	0.4-1.8	21 (2.2)	1.3-3.8
	Combination not classified by ATC	119 (3.2)	2.2-4.5	75 (3.7)	2.4-5.6	44 (2.1)	1.4-3.2
	Plants/herbal medicines	207 (5.5)	4.1-7.4	105 (5.4)	3.5-8.1	102 (5.8)	4.5-7.5
	Homeopathic agents	25 (1.2)	0.1-2.1	15 (1.2)	0.6-2.3	10 (1.4)	0.7-2.8
	Not identified/unknown	86 (2.4)	1.7-3.5	53 (2.3)	1.4-3.8	33 (2.7)	1.5-4.6
	Others	67 (2.4)	1.6-3.6	37 (2.2)	1.3-3.9	30 (2.7)	1.7-4.3
	Total	2,567 (100.00)		1,496 (100.00)		1,071 (100.00)	

95%CI: 95% confidence interval; ATC: Anatomical Therapeutic Chemical Classification.

Herbal medicines and/or plants corresponded to 5.5% (95%CI: 4.09-7.37), followed by combinations not classifiable by the ATC (3.15%; 95%CI: 2.15-4.46); additionally, roughly 2.2% represented antimicrobials for systemic use ( Table 3 ).

## DISCUSSION

It is noteworthy that there are no Brazilian studies on the use of medications addressing the prevalence of use of drugs to treat GID. Hence, this study is an unparalleled opportunity to observe the self-reported consumption profile of these drugs in Brazil.

This study showed the highest use of drugs for treating GID in women, regardless of the age range. In a national survey carried out only with retired individuals, higher use of drugs in the female sex was observed,^( 15 )^ corroborating the results obtained in this study. Nevertheless, a previous study pointed out that, in general, the difference in consumption of drugs between the sexes reduces with age.^( 16 )^

Considering this scenario, the more frequent use of drugs to treat GID in women might be explained by the higher risk of presenting with gastrointestinal symptoms in situations such as urinary tract infection, where abdominal pain is a characteristic symptom,^( 17 )^ and the menstrual cramps, which can often be perceived and treated as GID.^( 18 )^Consequently, this study strengthens this evidence and offers subsidies for public policies geared towards the rational use of drugs for GID in women.

In a study performed in France, the use of PPI was associated with polypharmacy and the high frequency of comorbidities in aged patients.^( 19 )^This result was also noted in this study by means of the more frequent use of drugs to treat GID among the aged, in those who presented with more than two comorbidities or in polypharmacy patients.

Considering that in this study we noted a higher prevalence of use of drugs for GID as the number of chronic diseases increased, and that PPIs were the most often mentioned drugs, it is possible to suggest a reflection in the sense that such drugs could have been utilized in an attempt to reduce possible gastric discomfort caused by the excessive use of drugs, in individuals with chronic diseases. Nevertheless, while observing, the use of drugs to treat GID in adults stands out with greater frequency in those who use one or more drugs.

A second hypothesis suggested is the increased use of these drugs in the elderly is due to the aging process, to which the progressive reduction of the individuals’ functional reserve takes them, affecting all gastrointestinal functions: motility, secretion of enzymes and hormones, production of saliva, digestion and absorption.^( 20 )^ A study showed that, even in healthy elderly persons, modifications had occurred in peristaltic movements and gastric emptying time compared to groups of young people.^( 21 )^

Additionally, within this context, the most utilized drugs to treat GIDs are classified as drugs for the alimentary tract and metabolism, and the most used drug was omeprazole, with a frequency 11.5 times higher than the second medicine on the list. A study done in Italy compared the use of treatments considered traditional with those considered alternative for GID^( 22 )^ also found a greater prevalence of use of PPIs. Scientific literature does not present studies of national surveys that show a prevalence of use of drugs to treat GID. Additionally, the studies found evaluate the consumption by means of another data collection method, or aim at consumption of PPI, or at a specific population.^( 3 , 4 , 6 , 10 , 11 , 19 , 22 )^

Considering the high prevalence of use of PPI found in this study, with a nationwide scope, it is important to point out the importance of the appropriate use of these drugs in the Brazilian context. Studies performed in developed countries pointed to gaps in prescription of PPI for patients after hospital discharge.^( 23 , 24 )^Within the realm of Primary and Secondary Care, some studies also showed inappropriate PPI prescription, taking into consideration the existing guidelines,^( 25 , 26 )^ even in developing countries, such as Mexico^( 27 )^ and Thailand.^( 28 )^

It should be noted that PPIs can cause adverse events and drug interactions, and should be used in a correct manner. This is why several studies highlight the importance of complying with the guidelines with the best evidence available for these prescriptions.^( 25 - 28 )^ A study conducted by Mousavi et al.,^( 29 )^presented evidence that drug conciliation at hospital discharge, besides the follow-up by the clinical pharmacist at the hospital, contribute towards the appropriate use of PPIs. In this way, by means of the results found in that study, it is possible to verify the high prevalence of the use of these drugs and to outline strategies, such as the incentive for use of guidelines for the prescription of PPIs, especially at Primary Care.

In light of these facts, the use of drugs for the treatment of GID may be linked to the indication of gastric protection caused by the use of polypharmacy, or the reduction of the physiological functionality, which is characteristic of the aging process.

As to the socioeconomic characteristics, in this study, the use of drugs for the treatment of GID was superior in those individuals who had a private health insurance. In a study conducted with data from the National Health Research (PNS - *Pesquisa Nacional de Saúde* ), it was noted that the prevalence of access and use of drugs for chronic non-communicable diseases in the Brazilian population is higher in the A, B, and C economic strata.^( 30 )^ Such a result corroborates the finding of the this study, as to the greater use of drugs to treat GID in individuals who have private health insurance, taking into consideration that these individuals have better economic conditions, and consequently, greater access to drugs.

About 10% of the drugs referred to treat GID do not have this indication. Within this context, this result could indicate lack of knowledge by part of the population as to the therapeutic indication, since the participants reported the use of antimicrobials and anxiolytics for the treatment of GID. Several authors have presented evidence that the knowledge of pharmacotherapy is associated with compliance, and consequently, there is a higher change of therapeutic success.^( 31 - 35 )^No studies were found evaluating the level of knowledge of the Brazilian population about the drugs. In this case, we point out the importance of performing nationwide studies that evaluate the knowledge of the population about pharmacotherapy, in order to direct strategies and public policies, with a view to therapeutic success.

Additionally, these results may also be explained by the complexity in classifications of the drugs according to their main indication, since the significant consumption of antimicrobials for systemic use may be a consequence of gastrointestinal infections, or of systemic infections that cause gastrointestinal symptoms.

This study has a few limitations inherent to the method used, such as the memory recall bias, since the information obtained was self-reported by the survey participants. Moraes et al.,^( 35 )^evaluated the agreement between a survey done at two instances in various groups, in which the difference was the time interval between the first and the second interview. In the group with the 14-day interval, it was noted that the Kappa value was 0.37 for occasional use of drugs, which fits into the classification of the drugs studied in this investigation. Moreover, the high number of different drugs, especially those not classified by ATC, hindered understanding of the use of the drugs studied. However, the national coverage of this study and its uniqueness are strengths, which qualify these results as tools to formulate strategies and promote rational use of drugs by the population.

## CONCLUSION

This study presented a diagnosis about the representativeness of the use of drugs for gastrointestinal disorders among the uses of other drugs, besides showing the sociodemographic profile of the Brazilian population that has more access to this type of drug (women, elderly, and patients with a private health insurance). Nevertheless, therapeutic success is not guaranteed only by access to treatment, but also by its appropriate use, and this study was able to raise hypotheses about this aspect. Longitudinal studies are required to test them, in order to fully understand the consumption of drugs for gastrointestinal disorders in Brazil, and to provide subsidies for the Brazilian government to draw up public policies that increase the access for those who need it, and promote the best use of these drugs.
