# Supplementary material for: Use of drugs for gastrointestinal disorders: evidence from National Survey on Access, Use and Promotion of Rational Use of Medicines
Source: Einstein (Sao Paulo). 2020 Aug 6;18:eAO5314. doi: 10.31744/einstein_journal/2020AO5314 (PMC7416649; doi:10.31744/einstein_journal/2020AO5314)
Supplement: Translation

# Translation: Utilização de medicamentos para distúrbios gastrintestinais: evidências da Pesquisa Nacional sobre Acesso, Utilização e Promoção do Uso Racional de Medicamentos

**Authors:** Lucas Borges Pereira, Ana Maria Rosa Freato Gonçalves, Camila Stéfani Estancial Fernandes, Andréia Turmina Fontanella, Priscila Maria Stolses Bergamo Francisco, Sotero Serrate Mengue, Rogério Boff Borges, Tatiane da Silva Dal Pizzol, Karen Sarmento Costa

**Affiliations:** 1 Brasil Faculdade de Ciências Farmacêuticas de Ribeirão Preto, Universidade de São Paulo, Ribeirão Preto, SP, Brasil.; 2 Brasil Departamento de Saúde Coletiva, Faculdade de Ciências Médicas, Universidade Estadual de Campinas, Campinas, SP, Brasil.; 3 Brasil Programa de Pós-Graduação em Epidemiologia, Faculdade de Medicina, Universidade Federal do Rio Grande do Sul, Porto Alegre, RS, Brasil.

**Keywords:** Uso de medicamentos, Assistência farmacêutica, Trato gastrointestinal, Inquéritos epidemiológicos, Farmacoepidemiologia, Inibidores da bomba de prótons, Polimedicação, Idoso, Política de saúde, Conhecimento do paciente sobre a medicação

## Abstract

**Objetivo:**

Estimar a prevalência de utilização de medicamentos para o tratamento de distúrbios gastrintestinais, segundo características demográficas, socioeconômicas e de saúde da população brasileira.

**Métodos:**

Trata-se de inquérito de base populacional, que entrevistou indivíduos residentes em municípios das cinco regiões do Brasil. A amostra deste estudo foi composta por 32.348 indivíduos de 20 anos ou mais de idade. Foi avaliado o perfil de utilização de medicamentos para distúrbios gastrintestinais entre as variáveis sexo, idade, plano de saúde, região e número de doenças crônicas. Analisou-se a frequência de indivíduos que declararam utilizar outros medicamentos, além daqueles já utilizados para tratamento de distúrbios gastrintestinais. Além disso, foram estimadas as frequências das classes medicamentosas utilizadas.

**Resultados:**

A prevalência de utilização de medicamentos para distúrbios gastrintestinais no Brasil foi de 6,9% (intervalo de confiança de 95% − 6,4-7,6), sendo maior no sexo feminino, entre pessoas acima de 60 anos de idade, naqueles que possuíam plano privado de saúde e tinham duas ou mais doenças crônicas. Observou-se que 42,9% dos idosos que utilizaram medicamentos para distúrbios gastrintestinais também eram polimedicados. Em relação às classes medicamentosas, 82% correspondiam a medicamentos para o trato alimentar e metabolismo, destacando os inibidores da bomba de prótons.

**Conclusão:**

A utilização de medicamentos para tratamento de distúrbios gastrintestinais foi significativa entre mulheres e idosos. Nesta faixa etária, o consumo pode estar vinculado à proteção gástrica causada por polimedicação. Este estudo representa oportunidade inédita para observar o perfil de consumo autorreferido desses medicamentos no Brasil e pode, portanto, subsidiar estratégias para promoção de seu uso racional.

## INTRODUÇÃO

Distúrbios gastrintestinais (DGI) são caracterizados por sinais e sintomas que acometem os tecidos e órgãos do trato gastrintestinal, como náusea, dor abdominal e queimação, originados a partir de uma doença de base.^( 1 )^Aproximadamente, 27% da população mundial tem constipação, um dos vários distúrbios que podem acometer o trato gastrintestinal.^( 2 )^Além disso, outros fatores podem levar ao aparecimento destes distúrbios, como a utilização de alguns medicamentos,^( 3 )^alimentação e estilo de vida.^( 4 )^Dessa maneira, a utilização de medicamentos para o alívio desses distúrbios torna-se frequente na população.

Os pacientes, em sua maioria, ao invés de investigar a doença de base ou fatores externos que podem originar os sintomas, preferem utilizar medicamentos que os aliviem. Um dos principais motivos é a facilidade na aquisição destes medicamentos, pois a maioria deles são medicamentos isentos de prescrição (MIP).^( 5 )^Além disso, em estudo realizado nos Estados Unidos, 34% dos médicos não estavam preocupados ou ignoravam os potenciais problemas que estes medicamentos podem causar ao paciente.^( 6 )^

Apesar dos MIP apresentarem características mais seguras em comparação com os demais medicamentos, muitos destes podem causar efeitos adversos leves a graves, principalmente quando se levam em consideração as particularidades fisiológicas e outras caraterísticas, como faixa etária, gravidez e utilização de mais medicamentos.^( 3 )^Além disso, aproximadamente 70% das mulheres grávidas no mundo sentem náusea, e 50% têm vômitos;^( 3 )^8% dos erros de medicação em asilos são causados por medicamentos para tratar DGI;^( 7 )^e 70,8% dos pacientes que são polimedicamentosos utilizam medicamentos de proteção gástrica.^( 8 )^Nesse cenário, é necessária atenção especial para com estas populações, com o objetivo de que os medicamentos para DGI sejam utilizados de maneira racional, tendo como propósito a redução dos riscos.

Em geral, a literatura científica apresenta estudos de utilização de medicamentos para tratar DGI que focam nas análises de subclasses desses medicamentos, como antagonistas de receptores H_2_e inibidores de bombas de prótons (IBP),^( 9 )^condições de saúde específicas,^( 10 , 11 )^e em populações específicas.^( 4 )^

No Brasil, o maior estudo de inquérito realizado sobre DGI foi o EpiGastro (2014) realizado na cidade de São Paulo. Neste estudo, entrevistaram-se 3.050 pessoas a respeito de informações que pudessem estar associadas à doença do refluxo gastresofágico e à dispepsia, bem como a maneira que esta população lidava com os sintomas. Observou-se que 13,6% não utilizavam medicamentos para tratar os sintomas, e 34,2% utilizavam medicamentos não prescritos por médicos.^( 11 )^No entanto, não há análise mais detalhada sobre os medicamentos para tratar DGI. Assim, são escassos estudos que avaliam, de maneira ampla, a utilização desses medicamentos em grandes populações.

Considerando a importância do tema e a escassez de estudos de base populacional, torna-se fundamental o desenvolvimento de inquéritos que tracem o perfil de utilização desses medicamentos nos diferentes segmentos populacionais, a fim de elaborar estratégias para sua utilização racional.

## OBJETIVO

Estimar a prevalência de utilização de medicamentos para o tratamento de distúrbios gastrintestinais, segundo características demográficas, socioeconômicas e de saúde da população brasileira; analisar a frequência de uso de outros medicamentos em concomitância aos medicamentos para tratamento de distúrbios gastrintestinais; e descrever as classes medicamentosas mais utilizadas para o tratamento de distúrbios gastrintestinais.

## MÉTODOS

Este estudo possui delineamento transversal de base populacional e foi realizado usando os dados oriundos da Pesquisa Nacional sobre o Acesso, Utilização e Promoção do Uso Racional de Medicamentos (PNAUM). A coleta de dados foi realizada entre setembro de 2013 a fevereiro de 2014, tendo sido entrevistados 41.433 indivíduos distribuídos em 245 municípios de todas as regiões do país; a metodologia completa desse inquérito está disponível em Mengue et al.^( 12 )^

Esta análise contempla as pessoas acima de 20 anos de idade residentes em áreas urbanas e capazes de se comunicar (n=32.348). A utilização de medicamentos para DGI foi avaliada por meio da pergunta: “nos últimos 15 dias, usou algum remédio para problemas no estômago ou intestino?”.

As análises foram realizadas utilizando o Stata 11.0 (Stata Corp LP, College Station, Texas, EUA), cujos procedimentos para análise de inquéritos populacionais incorporam aspectos da amostragem complexa, por meio dos comandos SVY *.* Foram realizadas análises estratificadas por duas faixas etárias: adulto (20 a 59 anos) e idoso (60 anos ou mais). O corte de 60 anos para classificação de idoso foi escolhido em acordo com o Estatuto do Idoso.^( 13 )^Para cada faixa etária, foi estimada a prevalência do uso de medicamentos para DGI, segundo características demográficas (sexo e região brasileira residente), socioeconômicas (posse de plano privado de saúde) e de saúde (número de doenças crônicas). A associação entre o uso de medicamentos para DGI e as variáveis independentes foi verificada por meio do teste χ^2^de homogeneidade, com nível de significância de 5%.

A fim de entender a influência da polifarmácia na utilização de medicamentos para tratar DGI, calcularam-se a frequência, a percentagem e o respectivo intervalo de confiança de 95% (IC95%) de indivíduos que declararam utilizar, além dos medicamentos para DGI, nenhum medicamento, um ou dois medicamentos, três ou quatro medicamentos, ou cinco ou mais medicamentos, sendo esta última categoria considerada, neste estudo, como indivíduo polimedicamentoso ou em uso de polifarmácia.^( 14 )^

Foram identificadas e estimadas as frequências, as percentagens e os IC95% das classes medicamentosas utilizadas segundo a primeira categoria do *Anatomical Therapeutic Chemical Classification* (ATC). As substâncias que não se enquadraram na classificação ATC foram categorizadas em: combinações não classificáveis pela ATC, sendo aqueles medicamentos compostos por combinações de fármacos com finalidade de atuar em mecanismos diferentes que convergem para o mesmo resultado, porém não são classificadas pela ATC (por exemplo, citrato de colina + betaína + metionina, indicados para tratar distúrbios metabólicos ou hepáticos, e cafeína + dipirona + orfenadrina, indicados para tratar alívio de dores associadas a contraturas musculares ou cefaleia tensional); planta/fitoterápico, composto por plantas, chás, garrafadas, tinturas e fitoterápicos; homeopáticos e, quando não puderam ser identificados, foram rotulados como “não identificados”, sendo estes os medicamentos digitados no questionário, mas que não podem ser identificados, provavelmente por erro na digitação do medicamento.

A PNAUM foi aprovada pela Comissão Nacional de Ética em Pesquisa, parecer 398.131, CAAE: 18947013.6.0000.0008. Todos os participantes assinaram um Termo de Consentimento Livre Esclarecido (TCLE).

## RESULTADOS

A prevalência de utilização dos medicamentos para DGI na população adulta brasileira foi de 6,9% (IC95%: 6,4-7,6), sendo maior entre as mulheres. Considerando-se os grupos etários, para ambos os sexos, a prevalência nos idosos foi o dobro daquela observada entre os adultos (14,4% *versus* 7,1% nas mulheres e 8,9% *versus* 4,2% nos homens, respectivamente). Também foi observada maior prevalência de uso desses medicamentos por aqueles que possuíam plano de saúde (8,5%; IC95%: 7,2-10,0%) em relação aos que não possuíam (6,5%; IC95%: 5,9-7,1) à época da pesquisa. O uso de medicamentos para tratar DGI foi de 16,2% entre os indivíduos que relataram a presença de duas ou mais doenças crônicas e de 3,8% (IC95%: 3,4-4,3) nos que não apresentavam doença crônica ( Tabela 1 ).

**Tabela 1:** Características da amostra e prevalência de utilização de medicamentos para o tratamento de distúrbios gastrintestinais na população brasileira segundo características demográficas, socioeconômicas e de saúde

Variável	Amostra n (%)	Prevalência de uso na população geral		Prevalência de uso em adultos (20-59 anos)		Prevalência de uso em idosos (60 anos ou mais)	
		
		%	IC95%	n (%)	IC95%	n (%)	IC95%
Total	32.348 (100,0)	6,9	6,4-7,6	23.283 (5,8)	5,2-6,4	8.995 (12,1)	10,9-13,4
Sexo		p<0,001		p<0,001		p<0,001	
Feminino	20.646 (53,7)	8,6	7,8-9,4	15.351 (7,1)	6,4-7,9	5.246 (14,4)	12,8-16,3
Masculino	11.702 (46,3)	5,0	4,4-5,8	7.932 (4,2)	3,5-5,1	3.749 (8,9)	7,7-10,2
Plano de saúde		p<0,001		p<0,05		p=0,0887	
Sim	6.156 (23,6)	8,5	7,2-10,0	4.139 (7,0)	5,8-8,6	1.999 (13,9)	11,3-16,9
Não	26.156 (76,4)	6,5	5,9-7,1	19.120 (5,4)	4,8-6,0	6.987 (11,4)	10,2-12,8
Região		p=0,1839		p=0,5159		p<0,003	
Norte	8.421 (6,7)	6,7	5,5-8,1	6.400 (6,4)	5,2-7,9	2.012 (8,5)	6,9-10,5
Nordeste	6.909 (23,4)	6,3	5,4-7,2	4.839 (5,7)	4,8-6,7	2.054 (9,4)	8,0-11,0
Sudeste	6.075 (47,5)	7,5	6,5-8,7	4.215 (6,0)	5,0-7,2	1.849 (13,2)	11,2-15,6
Sul	6.097 (14,7)	6,4	5,5-7,4	4.286 (5,0)	4,1-6,0	1.785 (12,4)	10,7-14,4
Centro-Oeste	4.846 (7,8)	6,8	5,8-7,8	3.543 (5,5)	4,4-6,7	1.295 (13,4)	11,1-16,0
Número de doenças crônicas		p<0,001		p<0,001		p<0,001	
Nenhuma	17.972 (60,3)	3,8	3,4-4,3	15.779 (3,7)	3,2-4,2	2.162 (4,9)	3,9-6,3
Uma	7.380 (21,0)	7,8	6,8-8,9	4.620 (7,5)	6,5-8,7	2.743 (8,6)	6,6-11,1
Duas ou mais	6.973 (18,7)	16,2	14,8-17,7	2.875 (15,1)	13,2-17,1	4.077 (17,4)	15,5-19,4

Valor de p do teste χ^2^. IC95%: intervalo de confiança de 95%.

No que se refere ao uso de outros medicamentos, além daqueles para o tratamento de DGI, a monoterapia ocorreu em apenas 16,3% (IC95%: 13,9-19,0) na população geral, sendo 20,7% (IC95%: 17,2-24,6) nos adultos e 7,3% (IC95%: 4,8-10,7) nos idosos. Entre os idosos, aproximadamente 43,0% dos que faziam uso de medicamentos para DGI também relataram uso de cinco ou mais medicamentos (polifarmácia) ( Tabela 2 ).

**Tabela 2:** Frequência de uso de outros medicamentos na população usuária de medicamentos para o tratamento de distúrbios gastrintestinais

Variável	População geral		Adultos (20-59 anos)		Idosos (60 anos ou mais)		Valor de p
		
	%	IC95%	%	IC95%	%	IC95%	
Número de outros medicamentos							<0,001
Nenhum	16,3	13,9-19,0	20,7	17,2-24,6	7,3	4,8-10,7	
1-2	35,5	32,2-38,9	41,8	37,5-46,3	22,4	18,7-26,5	
3-4	22,2	20,1-24,5	19,7	16,9-22,8	27,4	24,1-31,0	
5 ou mais	26,0	23,5-28,5	17,8	15,0-21,0	42,9	37,8-48,1	

Valor de p do teste χ^2^. IC95%: intervalo de confiança de 95%.

A tabela 3 mostra a distribuição das classes medicamentosas utilizadas para o tratamento de DGI. Cerca de 82,0% (IC95%: 79,0-84,3) dos medicamentos referidos pela população brasileira para tratamento de DGI eram aqueles classificados como medicamentos para o trato alimentar e metabolismo; destes, 75,5% eram medicamentos para tratar úlcera péptica e doença do refluxo gastresofágico (65,2% eram IBP); 6,8% antiácidos; 5,9% para desordem do funcionamento gastrintesintal; 4,2% propulsivos; e 4,0% antieméticos e antinauseantes.

**Tabela 3:** Distribuição das classes medicamentosas utilizadas para o tratamento de distúrbios gastrintestinais na população brasileira, segundo faixa etária

Primeiro nível ATC	Classes farmacológicas segundo ATC	População geral		Adultos (20-59 anos)		Idosos (60 anos ou mais)	
		
		n (%)	IC95%	n (%)	IC95%	n (%)	IC95%
A	Trato alimentar e metabolismo	1.981 (81,8)	79,0-84,3	1.156 (81,4)	77,7-84,6	825 (82,7)	78,7-86,1
J	Antimicrobianos para uso sistêmico	44 (2,2)	1,1-4,2	38 (3,0)	1,5-6,1	6 (0,4)	0,1-1,7
N	Sistema nervoso	38 (1,3)	0,8-2,1	17 (0,9)	0,4-1,8	21 (2,2)	1,3-3,8
	Combinações não classificadas pelo ATC	119 (3,2)	2,2-4,5	75 (3,7)	2,4-5,6	44 (2,1)	1,4-3,2
	Planta/fitoterápicos	207 (5,5)	4,1-7,4	105 (5,4)	3,5-8,1	102 (5,8)	4,5-7,5
	Homeopáticos	25 (1,2)	0,1-2,1	15 (1,2)	0,6-2,3	10 (1,4)	0,7-2,8
	Não identificados/ignorados	86 (2,4)	1,7-3,5	53 (2,3)	1,4-3,8	33 (2,7)	1,5-4,6
	Outros	67 (2,4)	1,6-3,6	37 (2,2)	1,3-3,9	30 (2,7)	1,7-4,3
	Total	2.567 (100,00)		1.496 (100,00)		1.071 (100,00)	

IC95%: intervalo de confiança de 95%; ATC: *Anatomical Therapeutic Chemical Classification* .

Os fitoterápicos e/ou plantas foram corresponderam a 5,5% (IC95%: 4,09-7,37), seguidos pelas combinações não classificáveis pela ATC (3,15%; IC95%: 2,15-4,46); além disso, cerca de 2,2% representaram a classe dos antimicrobianos para uso sistêmico ( Tabela 3 ).

## DISCUSSÃO

É importante salientar que não há estudos de utilização de medicamentos que abordem a prevalência de uso, a nível nacional, somente de medicamentos para tratar DGI. Dessa maneira, este estudo é oportunidade inédita para observar o perfil de consumo autorreferido desses medicamentos no Brasil.

Este estudo mostrou a utilização mais elevada de medicamentos para tratar DGI em mulheres, independente da faixa etária. Em inquérito nacional realizado somente com aposentados, observou-se utilização mais elevada de medicamentos no sexo feminino,^( 15 )^corroborando os resultados obtidos neste estudo. No entanto, estudo prévio apontou que, no geral, a diferença de consumo de medicamentos entre os sexos reduz com a idade.^( 16 )^

Considerando esse cenário, a utilização de medicamentos para tratar DGI mais frequente em mulheres pode ser explicada pelo maior risco de apresentação dos sintomas gastrintestinais em situações como infecção urinária, cuja dor abdominal é um sintoma característico,^( 17 )^e as cólicas menstruais que, muitas vezes, podem ser percebidas e tratadas como DGI.^( 18 )^Por conseguinte, esse estudo fortalece essas evidências e oferece subsídios para políticas públicas voltadas para utilização racional de medicamentos para DGI em mulheres.

Em estudo realizado na França, a utilização de IBP esteve associada à polimedicação e à alta frequência de comorbidades em pacientes idosos.^( 19 )^Este resultado também foi observado neste estudo por meio da utilização mais frequente de medicamentos para tratar DGI entre idosos, naqueles que apresentam mais de duas comorbidades ou nos pacientes polimedicados.

Considerando que, no presente estudo, observou-se maior prevalência de uso de medicamentos para DGI à medida que o número de doenças crônicas aumenta, e que os IBP foram os medicamentos mais citados, é possível sugerir uma reflexão no sentido de que tais medicamentos poderiam ter sido utilizados na tentativa de reduzir possíveis desconfortos gástricos causados pelo uso excessivo de medicamentos, em indivíduos com doenças crônicas. No entanto, ao observar a utilização de medicamentos para tratar DGI nos adultos, destaca-se maior frequência naqueles que utilizam um ou dois medicamentos.

Uma segunda hipótese levantada é a de que a utilização elevada destes medicamentos em idosos deve-se ao próprio processo de envelhecimento ao qual a redução progressiva da reserva funcional dos indivíduos leva, afetando todas as funções gastrintestinais: motilidade, secreção de enzimas e hormônios, produção de saliva, digestão e absorção.^( 20 )^Estudo mostrou que, mesmo em pessoas idosas saudáveis, ocorreram alterações nos movimentos peristálticos e no tempo de esvaziamento gástrico comparado a grupos de jovens.^( 21 )^

Ainda nesse contexto, os medicamentos mais utilizados para tratamento dos DGI são classificados como medicamentos para o trato alimentar e metabolismo, e o medicamento mais utilizado foi o omeprazol, com frequência 11,5 vezes maior que o segundo da lista. Estudo realizado na Itália comparou o perfil de utilização de tratamentos considerados tradicionais com os alternativos para DGI^( 22 )^e também encontrou maior prevalência de utilização dos IBP. A literatura científica não apresenta estudos de inquéritos nacionais que mostrem a prevalência de utilização de medicamentos para tratar DGI. Além disso, os estudos encontrados avaliam o consumo por meio de outro método de coleta de dados, ou objetivam o consumo de IBP ou em alguma população específica.^( 3 , 4 , 6 , 10 , 11 , 19 , 22 )^

Considerando a alta prevalência da utilização de IBP encontrada nesse estudo, cuja abrangência é nacional, é importante ressaltar a importância do uso apropriado desses medicamentos no contexto brasileiro. Estudos realizados em países desenvolvidos apontam lacunas na prescrição de IBP em pacientes após alta hospitalar.^( 23 , 24 )^No âmbito da Atenção Primária e Secundária, alguns estudos também apresentam prescrições inapropriadas do IBP, levando em consideração as diretrizes existentes,^( 25 , 26 )^inclusive em países em desenvolvimento, como México^( 27 )^e Tailândia.^( 28 )^

Ressalva-se que os IBP podem causar eventos adversos e interações medicamentosas e devem ser utilizados de maneira apropriada. Por isso vários estudos ressaltam a importância de seguir as diretrizes com as melhores evidências disponíves para a prescrição desses medicamentos.^( 25 - 28 )^Estudo realizado por Mousavi et al.,^( 29 )^apresentou evidências de que a realização da reconciliação medicamentosa na alta hospitalar, além do acompanhamento pelo farmacêutico clínico em ambiente hospitalar, contribui para a utilização apropriada dos IBP. Dessa maneira, por meio dos resultados encontrados nesse estudo, é possível verificar a alta prevalência da utilização desses medicamentos e traçar estratégias como o incentivo à utilização de diretrizes para a prescrição dos IPB, sobretudo no nível da Atenção Primária à Saúde.

Em vista desses fatos, a utilização de medicamentos para tratamento de DGI pode estar vinculada à indicação de proteção gástrica causada pelo uso de polifarmácia ou redução da funcionalidade fisiológica característica do processo de envelhecimento.

Considerando as características socioeconômicas, neste estudo, a utilização de medicamentos para o tratamento de DGI foi superior nos indivíduos que possuíam plano privado de saúde. Em estudo realizado com dados da Pesquisa Nacional de Saúde (PNS), foi observado que a prevalência do acesso e o uso de medicamentos para doenças crônicas não transmissíveis na população brasileira são maiores nas classes econômicas A, B e C.^( 30 )^Tal resultado corrobora os achados do estudo em questão, no que tange à maior utilização de medicamentos para tratamento de DGI em indivíduos que possuem plano de saúde privado, levando em consideração que tais indivíduos possuem maiores condições econômicas e, consequentemente, maior acesso a medicamentos.

Cerca de 10% dos medicamentos referidos para tratar DGI não possuem essa indicação. Nesse contexto, este resultado pode indicar falta de conhecimento por parte da população sobre a indicação terapêutica, já que os participantes referiram uso de antimicrobianos e ansiolíticos para tratamento de DGI. Vários autores apresentam evidências de que o conhecimento sobre a farmacoterapia está associado à sua adesão e, consequentemente, à maior chance de sucesso terapêutico.^( 31 - 35 )^Não foram encontrados estudos que avaliaram o nível de conhecimento da população brasileira acerca dos medicamentos. Nesse cenário, ressalta-se a importância da realização de estudos de abrangência nacionais, que avaliem o conhecimento da população sobre a farmacoterapia, a fim de nortear estratégias e políticas públicas, tendo em vista o sucesso terapêutico.

Além disso, este resultado também pode ser explicado pela complexidade de classificação dos medicamentos, de acordo com sua indicação principal, visto que o consumo significativo de antimicrobianos para uso sistêmico pode ser consequência de infecções gastrintestinais, ou de infecções sistêmicas que provoquem sintomas gastrintestinais.

Este estudo possui algumas limitações inerentes ao método empregado, como o viés recordatório, já que as informações obtidas foram autorreferidas pelos participantes do inquérito. Moraes et al.,^( 35 )^avaliaram a concordância entre um inquérito feito em dois momentos em vários grupos, cuja diferença era o intervalo de tempo entre a primeira entrevista e a segunda. No grupo com intervalo de 14 dias, observou-se valor de Kappa de 0,37 para medicamentos de uso ocasional, que se enquadra na classificação dos medicamentos estudados nesta pesquisa. Além disso, o elevado número de medicamentos diferentes, principalmente aqueles que não são classificados pela ATC, dificultou o entendimento na utilização dos medicamentos estudados. No entanto, a representatividade nacional deste estudo e sua unicidade são pontos fortes, que qualificam estes resultados como instrumentos para elaboração de estratégias de promoção para utilização racional de medicamentos pela população.

## CONCLUSÃO

Este estudo apresentou um diagnóstico sobre a representatividade da utilização de medicamentos para distúrbios gastrintestinais entre a utilização de outros medicamentos, além de mostrar o perfil sociodemográfico da população brasileira que mais acessa este tipo de medicamento (mulheres, idosos e pacientes com plano de saúde privado). No entanto, o sucesso terapêutico não é garantido somente com o acesso ao tratamento, mas também por sua utilização apropriada, e este estudo conseguiu levantar hipóteses sobre este aspecto. Estudos longitudinais são necessários para testá-las, a fim de compreender integralmente o consumo de medicamentos para distúrbios gastrintestinais no Brasil e fornecer subsídios para o governo brasileiro elaborar políticas públicas que aumentem o acesso para aqueles que necessitam e promovam a melhor utilização destes medicamentos.
